# The Nursing Supplement

**Published:** 1889-11-30

**Authors:** 


*The Hospital,*
NOVEMBER 30, 1889.

*Extra Supplement*

### The Nursing Mirror.

All Contributions for this Supplement should be addressed to the Editor, THE HOSPITAL, 139-140, Salisbury Court, Fleet Street, London, E.C., and should have the word "Nursing" plainly written in left-hand top corner of the envelope.

### En Passant.

SHORT ITEMS.—Mrs. Woodford Fawcett has started a nursing institution at 175, Tulse-hill, S.W.—The Duchess of Westminster has sent a donation of £50 to St. Mary's Nurses' Home, Plaistow.—The Municipal Council of Rome have decided to start a training school for nurses, of which the celebrated surgeon, Prof. Durante, shall be superintendent.—The late lady superintendent of St. John's Maternity Home has joined the Roman Catholic Church; being obliged to leave her post there, she entered Charing Cross Hospital as a nurse.—At Worcester Assizes, John Cuthbert, surgeon at Selly Oak Workhouse, was charged with committing an unlawful operation on a nurse in that institution, who in cross-examination discredited her previous evidence, and said her life for five years had been a living lie. The prosecuting counsel withdrew from the case, and the jury acquitted the accused.—Miss Coombe, matron of the Children's Hospital, Clifton, appeals for a Christmas treat for her patients and nurses.—Ten gentlemen have offered to give £10 each towards furnishing the nurses' home in connection with the Liverpool Southern Hospital.—Mrs. Levick has started a very successful branch to her home in Paris, where patients are cured of the morphia habit.

EVERY WOMAN A NURSE.—The following sentence is from last Saturday's *Lancet:* "It would be a real gain to society if the knowledge possessed by the trained nurse were made part of the education of every accomplished woman. Such knowledge would be worth a score of the 'ologies' in many a dark hour of domestic sorrow." What would the *Lancet* say if we wrote: "It would be a real gain to society if the knowledge possessed by the medical practitioner were made part of the education of every accomplished man. Such knowledge would be worth a score of 'ologies' in many a dark hour of domestic sorrow." What an outcry there would be about the popularisation of medical science! Then we should be told it was absurd for every man to devote four years of his life to obtaining a knowledge of medicine, and that even once obtained, without constant practice and keeping up with current discoveries, the knowledge would be useless. Quite true; so would it be absurd for every woman to devote three years of her life to nursing. What artists, and poets, and great singers we should have lost if Patti, Rosa Bonheur, Mrs. Browning, and others had had to give up three years of the best period of their lives to nursing. It would have been "a real gain to society," wouldn't it, never to have heard Patti sing, or to have read the "Sonnets from the Portuguese"? It is astonishing that a medical paper with such a name as the *Lancet* can be so ignorant of nursing matters, and utterly fail to see that nursing is not a drawing-room accomplishment, but a life's vocation. Besides, a nurse learns much that most women had better be ignorant of. It is necessary that some of the gentler sex should gain a knowledge of disease and sin; it is not desirable that all should do so. Let every woman attend the St. John's classes if she likes, but, in pity for the innocence which is the charm of so many young girls, do not propose that they should all be subject to the trial of three years in hospital. When every woman is taught nursing every man will be taught medicine, and then perhaps the *Lancet* will be happy. For our part we neither long nor look for such a devoutly not-to-be-wished-for consummation.

NURSES AND HEALTH.—In the Life of Miss Alcott is a severe passage, in which she condemns the two nurses who were attending to her father for living unhealthy lives—sitting over the fire and drinking tea, instead of taking proper exercise when off duty. It is indeed strange how many nurses, well versed in the laws of health, persistently disregard these laws, and overwork themselves, as though invalidism had no horrors for them. The constant drinking of strong tea, the staying indoors when leave is given for a walk, are both faults against which nurses should strongly strive. To quote George Herbert's lines, they should "know what's right, not only so, but also practise what they know."

OUR CORRESPONDENTS.—A soft parcel arrived at our office the other day which couldn't be books or papers, and therefore aroused interest. When opened it was found to contain a pretty Nightingale cloak sent by a correspondent whom we had advised to make these articles. Kind thoughts and kind letters are very cheering, and often make us regard those nurses we have never seen almost as personal friends. We are always glad to help our correspondents in any possible way we can, but unless it is absolutely necessary, we wish they would not ask for an answer by post, but be content to wait till the next number of THE HOSPITAL appears. The answer is often of interest to others besides the asker, and saves us the time necessary to write several private letters.

MANCHESTER DISTRICT WORK.—The annual meeting of Manchester and Salford Sick Poor and Private Institution was held this week, Mr. O. Heywood presiding. The annual report stated that in the year ended September last the institution was the means of supplying nursing to more than 3,450 patients. The private nursing had been satisfactorily conducted. The income received for nurses' services was £1,272, or £300 more than last year. There was a profit of £252, which went to support the district nursing. In connection with the association 3,400 patients had been visited during the year, entailing 91,000 visits. Mr. W. Rathbone, M.P., described the aims of the Queen Victoria Jubilee Institute for Nurses, and urged that if a union of those interested in the matter all over the kingdom could be brought about there would be a great improvement in nursing the poor in their own homes, because they would have the combined experience of the whole country collected and distributed for the benefit of the whole country.

ON THE CONTINENT.—It seems that at Strasburg the nursing is much like that we described lately as prevailing in Paris—religious sisters in cumbersome dresses, rough, untidy nurses, and a total absence of those small refinements and ornaments which make most English hospitals both pretty and comfortable. The correspondent of the *British Medical Journal* has been sending over accounts of German hospitals, but whereas many come in for condemnation of their nursing system, Prof. Von. Bergmann's hospital and that at Freidrichshain are highly commended. Not long ago a German doctor told an English nurse she should go to Vienna; there was no place like Vienna. He concluded, "When I wass dere I had sometimes as many as twentee dead bodies a day!" "And how about the nursing?" asked the nurse. "Oh! I do not know; I do not dink dere wass any. But, oh! it is a splendid place to learn!" This looks as though the science of surgery flourished better in Vienna than the art of nursing.

### The National Pension Fund for Nurses.

THE actuaries and accountants of the chief insurance companies have watched the progress of this fund with much kindly interest. Hospital authorities, officers, and nurses will read the following opinion of one of the leading insurance journals, the *Post Magazine*, with interest. W͏e shall hope to be able to find room for some similar articles recently published, and we will take this opportunity of thanking our *confreres* in the press for the very great assistance they have rendered in the establishment of the fund upon an assured basis. From the *Post Magazine*, 2nd Nov., 1889:

"We give the first report and accounts of the National Pension Fund for Nurses in another column, and they completely dispose of the violent, and we had almost said, unscrupulous attacks to which that institution was subjected by the *Lancet*. By the deed of settlement, it was necessary that a thousand proposals for annuity policies should be received before 1st January, 1890, in order that the society might be finally constituted. On 30th June last, the council was able to announce that the necessary number of applicants had been secured, and by that date 755 pension policies had actually been issued, besides 159 policies in the sickness branch. The receipts of the National Pension Fund from February, 1888, when it first started, to 30th June, 1889, amounted to £13,497 for annuities, (including £500 Mildmay Trust Fund, to be applied to annuities later on), £152 sickness premiums, and £25,758 donations and governors' subscriptions, in addition to which dividends were received to the amount of £1,483. The net result was that at the close of the year the funds in hand amounted to the handsome figure of £40,506, all thoroughly well invested, the only item in the balance-sheet of uncertain value being £75 for office furniture, and this will no doubt within a very short period be wiped out.

"The National Pension Fund for Nurses is the youngest of the institutions established under the Life Assurance Companies' Acts, but it has shown itself to be one of the most progressive and successful. To collect £13,497 as consideration for deferred annuities from a very limited portion of the population, and a class with perhaps a smaller average income than any other, is an unprecedented feat; and it shows that the organisers of the fund knew what they were about, and that there was urgent need for such an institution as they were bent on establishing. The National Pension Fund is not a charitable fund, but it is so planned as to be entirely supported by the contributions of its policy-holders. Nevertheless, it includes in its scheme a philanthropic element, as it lays itself out for donations from those interested in nurses and nursing institutions. The hospital nurse does not enter her calling with a view to pecuniary gain, but, in the majority of cases, does so because she feels that her services will do good to suffering humanity. She sacrifices worldly comfort for the sake of others, and it is therefore only right that those who have abundance of this world's goods should come to her assistance, and provide for her declining years, when she has been worn out by her arduous calling. The annual contributions of the nurses will, in themselves, provide the deferred annuities promised in the policies, and probably also secure additions by way of bonus. But all those who feel that nurses are a self-sacrificing class may, by contributing to the donation fund, assist in finding the little comforts which go far to cheer an old age after a life of toil. Already that fund amounts to nearly £26,000, principally owing to the princely generosity of Lord Rothschild, and Messrs. Anthony Gibbs and Sons, E. A. Hambro, and Junius S. Morgan; yet even that large sum will do but little for the ten thousand nurses who will before long form the membership of the fund, and we give our hearty good wishes to the effort which we understand is soon to be made to very largely increase this department of the National Pension Fund for Nurses."

### Ward Decoration.

NOVEMBER in the country is the busiest of months, by reason of the transplanting of shrubs and herbaceous plants, and the bedding out of spring blossoming flowers and bulbs, which must be completed ere the hard frosts set in. Among indoor things and window gardens there is not much doing beyond the arranging of bulbs, and the many varieties of sechems and saxifrages (always nice and green the whole year through) in the empty boxes which have during summer been gay with geraniums, lobelias, and other brilliant growths. Whoever plants snowdrops, hyacinths, and crocuses in window-boxes should be careful to place a slight coating of cocoanut fibre over the soil to protect the bulbs against severe frost, remembering to surround them when planting with sand to guard against damp, a more injurious enemy than cold. Bulbs of this kind may be got very reasonably from any nurseryman, and are seen to reward the purchaser for his outlay. Nothing is prettier in the early spring than the bright little blue variety of squill called Scilla Sibereca. This plant blossoms almost in the snow, and is perfectly lovely associated with snowdrop or crocus, being no higher than either of these, and of the colour of a May-day sky.

In order to grow hyacinths in glasses, as so many people like to do, they should be planted in the glass *just* above the water, which should, in the first instance, be tepid, then for six months shut away somewhere in the dark, in order that the roots may grow before the leaves start up. This is necessary to prevent weak, spindling foliage and disappointing bloom stalks appearing on the scene, to the grower's dismay later on. All plants which have flowered throughout the summer, and are now preparing for a winter's rest, should be carefully looked over, pruned, and trimmed generally by picking off all decaying leaves and buds, and then for a time diminish the supply of water, very little being necessary when the plants are in a drowsy condition, and almost all flowers require a rest some time or other during the year.

Many gardeners gradually cut off the supply of water altogether from fuchsias and other deciduous things, merely pruning and housing them carefully from the reach of frost until they show signs of coming to life again in the spring. Then they are taken out of the pots, the roots shortened and dressed ere they are put in fresh pots a size larger than before. Lilies, valotas, agapanthus, etc., are invariably treated in this way.

It is possible at this season to obtain plants somewhat cheaper than at any other, and the emptying of the parks and public pleasure grounds ought to enable Londoners to secure a supply very easily for next summer's use. Ferns about this time begin, in most cases, to take their annual rest, and do not therefore require so much water as usual, except in all instances where the foliage remains green and vigorous. On no account dry them off when they show no signs of fatigue; and should you possess window-boxes with plants of hardy ferns collected from native sources, do not deprive them of water during winter, even if they no longer have a green fruit on them, because in a natural state, though apparently withered to the roots, they are then supplied with more moisture than at any other time, and all this goes to provide strong luxurious growth for the following season. A lack of dampness in this state will only result in weakening the plants' growing capacities when spring comes back.

A north aspect for pot plants is to be avoided, ferns being the only things which approve of a shady situation for any length of time. If an east window can be secured all the better, because then the early rays of the sun fall on the flowers and they escape the furious heat of midday. A temperature of 55deg. is desirable at night throughout the winter, increased during the day some 10 or 15deg. more.

Ferns in wardian cases have a happy way of looking after themselves, and only need a little air now and then when the sides of the glass become too heavily coated with moisture. Careful attention to the watering, of course, is necessary, also that it be neither too much nor too little, and when the time comes for thinning out the contents of a bell glass, see that the ferns are not too suddenly plunged from a warm, damp atmosphere into a dry one. At all times avoid a rapid change of atmosphere, and in the selecting of ferns for hospital and indoor use I should be inclined to suggest the advice contained in the latter part of an ardent Scotch soldier's reply when asked what he would do if he were in command of Aberdeen, and he knew that the enemy were approaching from the north: "Ah should send oot scoots, and proceed with ca'tion."

### Everybody's Opinion.

[*Correspondence on all subjects is invited, but we cannot in any way be responsible for the opinions expressed by our correspondents. No communications can be entertained if the name and address of the correspondent is not given, or unless one side of the paper only be written on.*]

### NURSING AT NETLEY.

A SURGEON writes: I am rather surprised that you should insert in your annotations (HOSPITAL, Nov. 2nd,) what might lead the public to infer that there are no nursing sisters at the Royal Victoria Hospital, Netley, when, for the last 25 years at least, there has been a lady superintendent and several nursing sisters, in addition to a large number of probationers. All cases of a serious nature in military hospitals are most carefully nursed, and in cases, such as "enteric," two or three special orderlies are detailed to attend each case, so the patient is never, day or night, without attendance. Nursing sisters, one acting as superintendent, are serving at Aldershot, Dublin, Curragh, Shorncliffe, and other stations at home, and at Gibraltar, Malta, and Egypt abroad. At Netley and Woolwich there is a lady superintendent and nursing sisters. Some nursing sisters have called my attention to the "note" referred to, and I promised to write to you on the subject.—[We are quite aware of the excellent work done by the Army and Navy nursing sisters, but were merely expressing an opinion that only trained women should nurse our soldiers, many of whom, when sick, have to depend on the tender mercies of the orderlies. We were writing from special cases, two of which had lately come under our notice; one of typhoid, the other of pneumonia. In one case the orderly did not like to prevent his superior officer doing aught that he desired, the result being that the officer did foolish things and endangered his life. The subject bristles with difficulties, for this same officer utterly refused to be nursed by women.]

### LADIES AS DISTRICT NURSES.

SISTER RUTH writes: Will you kindly allow me space for a few words. I have read the correspondence on this subject with surprise and shame. Surely it is very unseemly to raise a controversy on the subject of the supposed superiority of one nurse over the other, speaking of one nurse as a "lady" and another as an "upper servant," and saying, too, how successful the "lady" nurses are with their patients, how fond they are of them, and what a great deal more good they do than the "upper servant nurse." I wish to remind them of the old adage, "Self praise is no recommendation"; also that greatness and humility go together. But the question is, "do ladies make good parish nurses?" I answer most emphatically, yes. But by lady, I mean those good, noble, and true women who have left wealth and luxury to don a nurse's dress, and who go into the homes of the wretched poor and attend them as woman to woman, who have no thoughts in their minds as to what their patients may or may not think of them. These noble women (and, thank God, there are many of them) do the work purely out of charity to their fellow-creatures, looking for no reward in this world. These, sir, are ladies. I know several nurses who are just as much devoted, who work on day by day patiently and cheerfully doing their duty, thinking nothing of risking their lives, if called upon to do so, for their patient's good. And these women, sir, are those spoken of so slightingly as belonging to the "upper servant" class. True, good women they are all the same, and who shall say that our Master does not think them quite as good (or better) than the other. "'Tis the mind that makes the man," so why not the woman too? I fear, sir, that I am trespassing too much on your valuable space, but I have tried to show that it depends on the woman whether it is the "lady" or the "servant" which make the best nurses. I do not like the term "lady nurse." A nurse is a nurse, be she a peeress or a peasant, so long as she receives a stipend, and I fear there are very few who do not: therefore where does the lady nurse come in?

### THE BEGINNING OF MILDMAY.

NURSE MAYALL writes: In the HOSPITAL of 2nd inst., I see the Mildmay Park Nursing Institution, the Green, Stoke Newington, states it was established in 1873. If this be the correct date, what was the same institution supposed to be in the year 1869? I entered it as a trained nurse then from St. Bartholomew's, and worked for the same hospital nearly four years, it being in another part of the Mildmay-road, from where we removed to after. Mrs. Lustcomb was the matron whom Miss Bell succeeded, the Rev. W. Pennyfather being then alive. I hold good proof of the dates I quote above.

### VOTING BY PROXY AT BRIGHTON.

A SUBSCRIBER writes: I enclose a cutting from our daily paper advertising for a house surgeon for the Sussex County Hospital, by which you notice that ladies only are allowed to vote by proxy at the election. Can you inform me if such a system is followed in any other hospital? for if so, the sooner it is done away with the better, as by it the chances are that the successful candidate is chosen by his influence with the fair sex and not for his professional skill. It is also unjust to male subscribers, as the election takes place at hours when business men are engaged, inconvenient for some and impossible for others to attend; the male invalid subscribers are also deprived of any voice in the election. Equal rights, or the exercise of them—for women are well enough, but not if superior to men—in such grave matters as deciding the respective merits of doctors. It would be much better in a large establishment like the Sussex County Hospital to leave the selection to the staff of medical men attached to the institution, and in all probability the best man would be elected; much unnecessary expense saved to the candidates in printing testimonials, proxy letters, etc., and last, but not least, carriage hire to enable him to visit and curry favour with the dames of Brighton—an influential body, as lately Sir Robert Peel found out to his cost.

### THE SCANDAL AT HOPE INFIRMARY.

LUNACY writes: Under the above heading a correspondent, signing herself "Justice," asks several questions about the rats being placed in the late matron's bed. The purport of the appearance of "Justice" in print appears to be the ventilation of her opinion that the story of a lunatic being the culprit is a "blind," and that there was someone besides who was not discovered. If it were not that the accusation of neglect of duty is thrown out, the letter is scarce deserving of notice; but, being one of the attendants personally responsible for the care of the insane in the Salford Union Infirmary, I think a little explanation will not be amiss respecting the very regrettable incident referred to. In this institution there are a number of weak-minded and epileptic patients, male and female, classed as harmless, who reside in pavilions set apart for them, but many of whom are daily employed in all parts of the hospital in such work as is best suited to their health and mental capacity; some of the males as messengers, etc., and some of the females in the central kitchen, the pantries, the sewing-room, the laundry, and other places about the administrative pavilion. To one of these females the late matron had applied the epithet, so the patient said, of a "fat, squinting woman," which so incensed her (being thus afflicted in her eyes) that it was not considered advisable to let her out of her ward for fear of her retaliating on the matron. This patient, an epileptic, had made a discovery concerning one of the ward doors that had escaped attention, which was that it had a defective bolt and could be opened without the key (this she afterwards showed to the infirmary committee with pride), and she appears to have taken advantage of it by quietly slipping out unnoticed and going to the matron's bedroom, which her previous knowledge of the building enabled her easily to enter. It was only a few months before this that another female epileptic assaulted the late matron in the laundry by striking her on the face with a dead rat because she said she had been called by her "a ticket-of-leave woman," she, the patient, having been in prison. The lunacy commissioner, on his last visit here, made a report of this occurrence, and these facts came to the knowledge of the guardians. Knowing the character of the epileptics, I am thankful that nothing even more serious occurred.

### A REGISTRY FOR NURSES.

NOT IN WANT OF WORK would be pleased to undertake the superintendence of a well organised association for the benefit of nurses; but fears that a registry office is not a suitable thing, nor would it command the confidence of the public, which is so essential for the prosperity of such a work, but will be very glad to attend a meeting on the subject, if called by the Hospitals Association.

### Notice.

### EXHIBITION OF NURSING UNIFORMS.

THE ward kindly offered for this exhibition at St. Thomas's having proved unsuitable on inspection, the authorities of Charing Cross have generously put their board-room at our disposal for Monday and Tuesday, December 9 and 10, where the exhibition will be held. All nurses in uniform will be admitted free; those out of uniform and their friends will be charged 1s. each, and the net proceeds will be equally divided between the Pension Fund and Charing Cross Hospital. On Monday, the exhibition will be open from 10 a.m. until 8 p.m.; on Tuesday, from 11 a.m. till 6 p.m. Tickets will be obtainable at the doors.

### Lectures.

A COURSE of five lectures, free to the public, as required by the will of Mr. Brown, will be delivered by Victor Horsley, Esq., M.B., F.R.S., in the theatre of the University of London, Burlington-gardens, W., on the afternoons of November 29th, and of December 2nd, 4th, 6th, and 9th, commencing each day at 5 o'clock. Subject: "Fractures of the Skull and Compression of the Brain."

### Appointments.

[It is requested that successful candidates will send a copy of their applications and testimonials, with date of election, to THE EDITOR, The Lodge, Porchester-square, W.]

GREAT YARMOUTH HOSPITAL.—Miss S. C. Wiltshire has been appointed matron of this hospital. Miss Wiltshire was for a few months at the London Hospital, and has taken temporary matron's duty at Great Yarmouth. Considering the reasons for which the late matron left, it is a pity the committee have not appointed an older and more experienced nurse. Still we wish Miss Wiltshire every success in ruling over the six nurses and five servants who will be under her sway. Forty beds can be made up, but the average occupied is 29. There is a resident house surgeon.

JAFFRAY HOSPITAL.—Miss Elizabeth Wemyss has been appointed matron of this hospital near Birmingham. Miss Wemyss trained at the Middlesex, and spent some time there taking holiday work as sister and superintendent, thus gaining a knowledge of housekeeping and general management. Lately Miss Wemyss has been engaged in private nursing, but, like most nurses, now desires a return to institution life. We wish her all success.

NETLEY HOSPITAL.—Miss Helen C. Norman has been appointed lady superintendent of the Royal Victoria Hospital, Netley, in place of Mrs. Deeble, resigned.

ROYAL HOSPITAL FOR DISEASES OF THE CHEST.—As we briefly announced last week, Mr. Harrold has been appointed to the secretaryship of this hospital in place of Mr. Austin. Mr. Harrold holds excellent testimonials from Lord Balfour, Dr. Cayley, Major Christie, and others. Mr. Harrold has been four and a half years assistant secretary at the London Fever Hospital, and was once acting secretary to Mr. Channing, M.P., and formerly private secretary to Mr. Magniac, M.P. Mr. Harrold hopes to greatly improve the financial affairs of the Chest Hospital.

ST. LEONARDS CONVALESCENT HOME.—Miss Annie Briggs has been appointed lady superintendent of the Convalescent Home for Poor Children at St. Leonards. Miss Briggs trained at Westminster Hospital, staying there for five years ; she then went to superintend the Bangor Nursing Institute, in Wales. This institute she raised from the lowest ebb to the very highest state of efficiency. There were upwards of 100 candidates for the post at St. Leonards, but Miss Briggs held such excellent testimonials, they gained her the day. Her new duties commence on December 30.

### Notes and Queries.

TO CORRESPONDENTS.—1. Questions may be written on post-cards. 2. Advertisements in disguise are inadmissible. 3. In answering a query please quote the number. 4. If a private answer is desired, a stamped addressed envelope must be forwarded.

NOTICE.—We must really ask our correspondents to be more particular in enclosing name and address. Constantly we receive queries or letters with no name attached.

### Queries.

(25) *Convalescent Home.*—Can anyone tell me of a home where a girl recovering from anæmia could be received free of expense. The girl is in very poor circumstances. *Marjorie.*

(26) *Chiropody.*—Nurse Janet wishes to know where she can be instructed in the above.

(27) *Dictionary.*—Which is the best medical pronouncing dictionary, and the best book on physiology? *Wing.*

### Answers.

*Nursing at the Cape.*—Miss Mollett, of Chelsea Infirmary, is advertising for eight nurses to go to the Transvaal, and Miss Watkins, of Nailsworth, Gloucester, is advertising for a lady midwife for Kimberley. *Helen* should watch our advertisement columns.

*Nina.*—Kola-nut paste makes a kind of chocolate, which is said to be what is called an "antitriptic," or preventer of tissue waste. It is therefore, assuming this description of it to be correct, a "nutrient" of high value. We do not believe that it "effectually drowns the craving for strong drink," though by its sustaining and stimulating properties it may help an inebriate to resist temptation to some extent. It ought to be obtainable through any ordinary chemist, or directly from Messrs. Thomas Christie and Co., of London.

### Presentations.

AT Birmingham New Infirmary on November 19th, the matron was presented by her sisters, nurses, and probationers with a pair of candlesticks, inkstand, and penholders, all very handsomely embossed.

### Vacancies.

THE following vacancies are announced :

### Matrons.

Sheffield Union (assistant), £35.
Stafford Infirmary, £60.
West of England Eye Infirmary, £40.

### Sisters.

Swansea General Hospital (two), £25.

### Nurses.

Albert Hospital, Devonport, £25.
Amersham Union, £25.
Bedfordshire Institute, £26.
Belfast Nurses' Home.
Belgrave Hospital for Children.
Blackheath Institute, £20.
Brompton Consumption Hospital, £25.
Buckinghamshire Infirmary, £25.
Canterbury Institute (three).
Cheltenham Nursing Institute.
Chichester Infirmary (head).
Devon and Exeter Hospital, £24.
Eastbourne Nursing Institution.
Frome Nurses' Home, £22.
Hertford Infirmary, £20.
Highbury Institute.
Kent Nursing Institution, £30.
Kidderminster Infirmary, £25.
Leicester Institution, £25.
London and Brighton Association.
Mayford Industrial School, Woking, £24.
Newcastle City Hospital, £25.
Newlands, Ryde (three).
Newport Institute.
Nottingham Institution, £25.
Nursing Institution, 96, 97, 98, Wimpole-street, London, W., Mr. Wilson has several vacancies for thoroughly trained nurses.
Paddington Green Children's Hospital.
Peterborough Infirmary, £20.
Royal Albert Hospital, Devonport.
Scarborough Nurses' Institute.
Sheffield Nurses' Home.
Southport Nurses' Home (several).
South-Western Fever Hospital, £30
St. John's Institute, Southport, £22.
St. Leonard's Nurses' Home.
St. Saviour's Infirmary, £20.
Stratford-on-Avon Hospital, £20.
West London Hospital, £24.
Wimbledon Convalescent Hospital, £20.
Workhouse Infirmary Nursing Association.

### District Nurses.

Bond End, Settle, £60.
5, Charlotte-street, Edinburgh.
District Home, Bolton.
Trinity Parsonage, Derby, £50.

### Probationers.

Central London Ophthalmic Hospital.
Barnet Cottage Hospital.
Batley Hospital.
Dawlish Cottage Hospital.
H. M. Hospital, Stepney.
Isle of Man Hospital.
London Fever Hospital.
Lowestoft Hospital.
North-West London Hospital.
Swansea General Hospital (two).

### Amusements and Relaxation.

### Third Quarterly Word Competition Commenced October 5th, 1889, ends December 28th, 1889.

Three Prizes of 15s., 10s., 5s., will be given for the largest number of words derived from the words set for dissection.

Proper names, abbreviations, foreign words, words of less than four letters, and repetitions are barred ; plurals, and past and present participles of verbs, are allowed. Nuttall's *Standard* dictionary only to be used.

N.B.—Word dissections must be sent in WEEKLY, arranged alphabetically, with correct total affixed.

The word for dissection for this, the *Ninth* week of the quarter, being "T U R K E Y S."

### Results of Seventh Week.

| Names. | Nov. 21st. | Totals. | Names. | Nov. 21st. | Totals. |
|---|---|---|---|---|---|
| Winifred | — | 672 | Sunshine | 93 | 907 |
| Adelaide | — | 646 | Electrician | 88 | 1032 |
| Nettie Vance | — | 757 | H. C. | — | 562 |
| Amos | — | 596 | Reldas | 99 | 1075 |
| F. W. P. H. | 101 | 1060 | Rover | — | 776 |
| Reyot | — | 369 | Qu'appelle | 90 | 886 |
| Ellice | 91 | 1089 | Beryl | — | 188 |
| A. S. K. | — | 275 | Lightowlers | 90 | 1122 |
| Neptune | 94 | 1055 | Hercules | — | 12 |
| Ecila | 101 | 1149 | Jenny Wren | 71 | 720 |
| Tinie | — | 17 | Night Watch | — | 346 |
| Amicus | — | 946 | Oakenrod | 91 | 1122 |
| Juvenis | — | 539 | Embryo | — | 385 |
| Jumps | 100 | 1154 | Gypsye | 91 | 967 |
| Sister | — | 438 | Leo | — | 676 |
| M. W. | 100 | 1099 | Pearl | — | 299 |
| Esperance | 101 | 1104 | Africando | — | 171 |

### Notice to Correspondents.

N.B.—All letters referring to this page which do not arrive at 139 and 140, Salisbury-court, London, E.C., by the *first post on Thursdays*, and are not addressed PRIZE EDITOR, will in future be disqualified and disregarded.

